# Synthesis of the System Modeling and Signal Detecting Circuit of a Novel Vacuum Microelectronic Accelerometer

**DOI:** 10.3390/s90604104

**Published:** 2009-05-27

**Authors:** Dongling Li, Zhiyu Wen, Zhongquan Wen, Xuefeng He, Yinchuan Yang, Zhengguo Shang

**Affiliations:** 1 Key National Lab of Novel Micro/Nano Devices and System Technology, Chongqing 400030, China; 2 National Center for International Research of Micro/Nano System and New Materials Technologies, Chongqing 400030, China; 3 Microsystem Research Center, and College of Optoelectronic Engineering Chongqing University, Chongqing 400030, China

**Keywords:** vacuum microelectronics, accelerometer, closed-loop control, weak current detection

## Abstract

A novel high-precision vacuum microelectronic accelerometer has been successfully fabricated and tested in our laboratory. This accelerometer has unique advantages of high sensitivity, fast response, and anti-radiation stability. It is a prototype intended for navigation applications and is required to feature micro-g resolution. This paper briefly describes the structure and working principle of our vacuum microelectronic accelerometer, and the mathematical model is also established. The performances of the accelerometer system are discussed after Matlab modeling. The results show that, the dynamic response of the accelerometer system is significantly improved by choosing appropriate parameters of signal detecting circuit, and the signal detecting circuit is designed. In order to attain good linearity and performance, the closed-loop control mode is adopted. Weak current detection technology is studied, and integral T-style feedback network is used in I/V conversion, which will eliminate high-frequency noise at the front of the circuit. According to the modeling parameters, the low-pass filter is designed. This circuit is simple, reliable, and has high precision. Experiments are done and the results show that the vacuum microelectronic accelerometer exhibits good linearity over -1 g to +1 g, an output sensitivity of 543 mV/g, and a nonlinearity of 0.94 %.

## 1. Introduction

High precision accelerometers find many applications such as acoustic measurement, seismology and navigation. Micro-machined accelerometers have been developed with different working principles [1-3]. However, with the fast development of modern science and technology, the micro accelerometers are required to have better performance, such as high precision, good stability and linearity, low power consumption, high temperature resistance, etc. So exploration and research on high performance micro accelerometers is still a hot research topic. For high performance, two aspects are focused on. One is the exploration of new principles and novel structures, and the other is the design of an appropriate signal detecting circuit, which will directly influence the characteristics of the accelerometer system.

Since the vacuum microelectronic technology was proposed in 1988, the possibility of producing a high precision, good performance vacuum microelectronic sensor has been actively explored. The study of vacuum microelectronic sensors started in 1991, and since then many new types of sensors have appeared [4], such as pressure sensors, accelerometers, magnetism sensors and image sensors. Vacuum microelectronic devices are designed based on field emission theory, and the sensing part works under vacuum conditions. It utilizes the cold cathode to emit electrons, the emission current density is mainly determined by the electric field density near the tip array, which is exponential to the distance between the anode and the cathode tip array. Compared to other common well-developed accelerometers, vacuum microelectronic accelerometer has unique advantages of anti-radiation, small size, high sensitivity and the compatibility for fabrication process with integrated circuits (IC). It is widely required in applications such as small satellites, navigation, dexterous projectiles, tactical missiles and industrial automatic control [5].

The signal detecting circuit is another essential part for high precision micro accelerometers, and considerable research work has been done in this area too. Analog Devices Company has designed a modulation and demodulation circuit for capacitive accelerometers since the 1990s [6]. Now it is integrated with an accelerometer on a chip, and the accelerometer has good performance. Stanford Integrated Circuit Laboratory developed a high precision tunneling accelerometer and the corresponding closed-loop control circuit in 1998. This accelerometer can attain micro-g resolution [7]. Nowadays, several groups have begun to study the influence of signal detecting circuits on the characteristics of accelerometer systems. Based on the system modeling, they do analysis of the system using Matlab software, and then attain appropriate circuit parameters to instruct the design of signal detecting circuits [8-9].

The objectives of the present research are to design a signal detecting circuit for a high precision vacuum microelectronic accelerometer, which will ensure good linearity, high sensitivity and fast response of the accelerometer system. In this paper, first the structure and working principles of a vacuum microelectronic accelerometer are introduced, and then the mathematical model is established. We plot and discuss some simulation results of the dynamic performance and stability of the system, and confirm the circuit parameters. The electrostatic force balance technology is adopted in the circuit, and the application range of vacuum microelectronic accelerometer is greatly extended.

## 2. Structure and Working Principle

**Figure 1.**
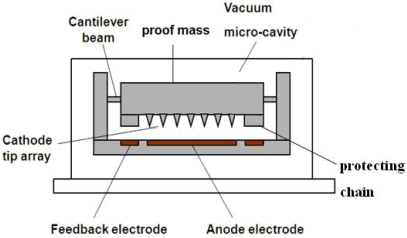
Structure diagram of vacuum microelectronic accelerometer.

The structure diagram of a vacuum microelectronic accelerometer is illustrated in Figure 1. The mechanical components comprise four cantilever beams, a proof mass and a micro-silicon field emission tip array. The electrodes include a cathode, an anode and a feedback electrode. Meanwhile, the protecting chain is designed. It will prevent the damage of the tip array and realize over loading self-protection. When the acceleration exceeds the measurement range, the anode will contact with the protecting chain, and avoid the collision between the anode and the tip array.

**Figure 2.**
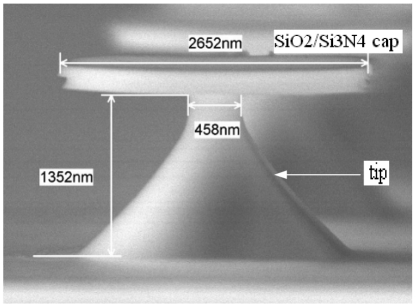
The SEM diagram of the single tip.

This accelerometer has been designed and fabricated. The dimensions of the accelerometer are obtained. Figure 2 is the SEM diagram of single tip. The bottom pyramid is the tip, and the top plate is the SiO_2_/Si_3_N_4_ cap protecting the tip from being eroded. Finally, the cap will be removed after the tip acuity. When a big enough DC voltage is added between the tip and the anode electrode, the tip will emit electrons under high electric field.

The vacuum microelectronic accelerometer works in electrostatic force balance mode. The working principle is that by applying a forward bias voltage between the anode and cathode, when the bias voltage is large enough, the tip array begins to emit electrons under high electric field, and then the electrons form a diode forward current. When the bias voltage is constant and there is an acceleration acting on the accelerometer, the proof mass will produce a displacement, and result in the change of emission current. Using current detecting circuit and electrostatic negative feedback system can make the proof mass maintain the balance position, and then the acceleration is obtained by measuring the output voltage.

## 3. Mathematical Model and System-Level Analysis

### 3.1. The Mathematical Model

Matlab was used to build the mathematical model of the vacuum microelectronic accelerometer. The model is composed of different function blocks based on Laplace transforms. In general, a vacuum microelectronic accelerometer with a feedback control system is not a linear system. Assumptions and approximations are used to linearize the system.

#### 3.1.1. The Sensing Part

The proof mass is the sensing part of the accelerometer. It can be considered as a suspended mass-spring-damping system [10]. Using Laplace transforms, the dynamic performance of the proof mass can be expressed as:

(1) $$G_{1}(s)=\frac{\Delta x}{m\Delta a}=\frac{1}{ms^{2}+bs+k}$$

where *m, b*, and *k* represent the mass, damping coefficient, and spring constant of the proof mass, respectively. *Δa* is the external acceleration, and *Δx* is the displacement of the proof mass. It is a typical two-order system.

#### 3.1.2. The Change of Displacement to the Current Part and the Circuit Part

According to field emission theory and the prior modeling results [11], the emission current is exponentially proportional to the change of the displacement of proof mass, so theoretically the vacuum microelectronic accelerometer can attain high sensitivity. The relationship between the displacement of the proof mass and the emission current is approximately given by:

(2) $$I=I_{0}\exp(-\alpha \Delta x)$$

where *I_0_* is the static emission current, *α* is a constant referring to the structure of vacuum microelectronic accelerometer. As we can see, this is not a linear relationship, so a linearization is required. Since the displacement of the proof mass Δx is very small, Equation (2) can be developed by Taylor series and as:

(3) $$I=I_{0}(1-\alpha \Delta x)$$

The emission current goes through I/V conversion circuit, and compared with a reference voltage *V_ref_*. Then the current is amplified by a main amplifier. The output voltage can be expressed as follows:

(4) $$V=K\cdot R\cdot (I-I_{0})=I_{0}\cdot R\cdot K\cdot \alpha \cdot \Delta x$$

*R* is the equivalent resistance of I/V conversion, and *K* is the amplitude of the amplifier. Therefore, the nonlinear relationship is linearized. Otherwise, a low-pass filter with transfer function *H_1_(s)* is needed in the circuit.

#### 3.1.3. The Feedback Control Loop

For an electrostatic force balance accelerometer, the effect of a feedback control loop is to give a static force which is opposite to the acceleration, and make the proof mass maintain the balance position. Ideally, when the acceleration is zero, the proof mass would be at the balance position and the distance between the anode and the cathode is *x_0_*; if the acceleration is not zero, the proof mass would produce a small distance *Δx*. In the event that the gain of the closed loop is big enough, there would be a negative electrostatic force produced by the feedback voltage. Then the force loads to the feedback electrode, and makes the proof mass return to the balance position, so in the closed loop control, the proof mass will move in a very small distance, and the linearity of the system is improved.

**Figure 3.**
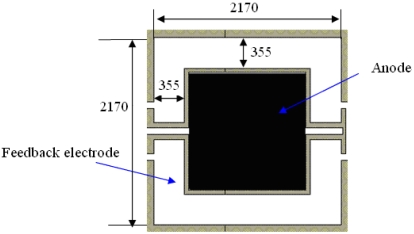
Bottom electrode of vacuum microelectronic accelerometer.

Figure 3 shows the bottom electrode of the vacuum microelectronic accelerometer. The feedback electrode can be seen as four approximately rectangular plates, and the dimensions (μm) are described in the figure. Referring to Figure 1 and the working principle, the actuator can be seen as a parallel capacitor [12]. Compared to *x_0_*, the dimension of the feedback electrode is much bigger and the marginal effect can be neglected. So the feedback electrostatic force *F_f_* can be described as:

(5) $$F_{f}==\frac{\varepsilon_{0}s{\left(V_{dc}+V_{ac}\right)}^{2}}{2{\left(x_{0}+\Delta x\right)}^{2}}$$

where *V_dc_* is the DC deflection voltage ensuring the original working state and *V_ac_* is the output voltage which is proportional to the acceleration. *ε_0_* is the vacuum dielectric constant and *s* is the overlap area of the feedback electrode to the cathode.

Since *V_dc_* (24V) is much bigger than *V_ac_* (several mV), and *Δx* is much smaller than *x*, the electrostatic force can be given by:

(6) $$F_{f}=\frac{\varepsilon_{0}s}{2{x_{0}}^{2}}({V_{dc}}^{2}+2V_{dc}\;V_{ac})$$

The first item is used to set the operation point, and the second item is related to the feedback force applied to the proof mass. At small signal analysis, Equation (6) can be simplified as:

(7) $$F_{f}=\frac{\varepsilon_{0}s}{{x_{0}}^{2}}V_{dc}\;V_{ac}=K_{f}\;V_{ac}$$

**Figure 4.**
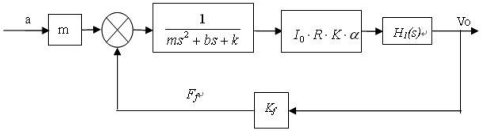
Transfer function diagram of vacuum microelectronic accelerometer system.

So we obtained the overall transfer function diagram of the system, as shown in Figure 4.

#### 3.1.4. The Linearity of the System

As analysis above, when the acceleration acts on the accelerometer, the proof mass will move beyond a balance position. According to force balance theory, the relationship can be given by:

(8) $$k\Delta x=ma-F_{f}$$

where *m* is the mass and *a* is the external acceleration. For closed-loop control system, the displacement Δx is very small, so *F_f_* » *kΔx*. We can obtain: *ma* = *F_f_*, and the relationship between output voltage and acceleration can be expressed by Equation (9), which illustrates that the vacuum microelectronic system has good linearity:

(9) $$a=\frac{F_{f}}{m}=\frac{K_{f}\;V_{ac}}{m}$$

### 3.2. The System-Level Analysis

Based on the linearized mathematical model, the system can be simulated and the characteristics of the accelerometer can be evaluated. The system-level analysis can effectively instruct the design of signal detecting circuit, and enhance the performance of the accelerometer system. Under current encapsulation, the vacuum micro cavity is not under an absolute vacuum condition, so a damping coefficient is exists. It is determined by the structure of vacuum microelectronic accelerometer. When the structure is determined, all the design parameters are obtained. Table 1 shows the structure parameters of vacuum microelectronic accelerometer.

**Figure 5.**
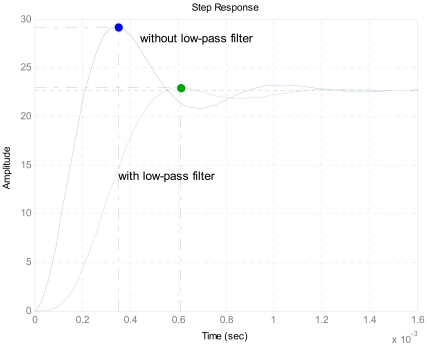
Step response of the accelerometer system with and without low-pass filter.

Figure 5 shows the step response of the system. It illustrates that before adding the low-pass filter, the overshoot is 28.7 %, and the adjusting time is 1.09 ms; after the low-pass filter is added, the overshoot drops to 0.59 %, and the adjusting time becomes 0.97 ms. By calculation the damping ratio of the system is 0.73 and the natural frequency is 674 Hz. It is proven that by adding a proper low-pass filter, the dynamic performance of the accelerometer is remarkably improved and the system has good performance. Here, the low-pass filter is a typical 2-order low-pass filter, and the cut-off frequency is 600 Hz. The transfer function of low-pass filter is given by Equation (10). It can be easily realized by a simple circuit using resistors, capacitors and amplifiers:

(10) $$H_{1}(s)=\frac{1}{5\times 10^{-9}s^{2}+2.0\times 10^{-4}s+1}$$

**Figure 6.**
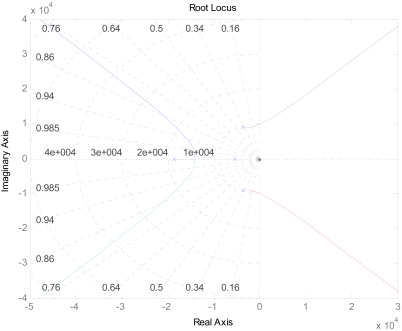
Root locus of accelerometer system

The root locus of the system is shown in Figure 6. It demonstrates that after adding a low-pass filter, two poles on the left side of s-plane are added. The dynamic performance of the accelerometer is improved, but the relative stability is decreased [13]. All the poles will be located on the left side of the s-plane when the gain is less than 8.5×10^3^, and it will be easily accomplished in the actual circuit.

## 4. Design of Signal Detecting Circuit

**Figure 7.**
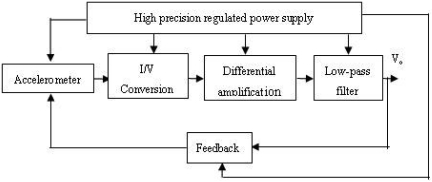
Structure diagram of detecting circuit.

In order to improve the linearity characteristic of the output, the signal detecting circuit works on close-loop mode, as shown in Figure 7. It mainly includes I/V conversion circuit, differential amplifier, low-pass filter, feedback control loop and high precision regulated power supply.

### 4.1. I/V Conversion Circuit

**Figure 8.**
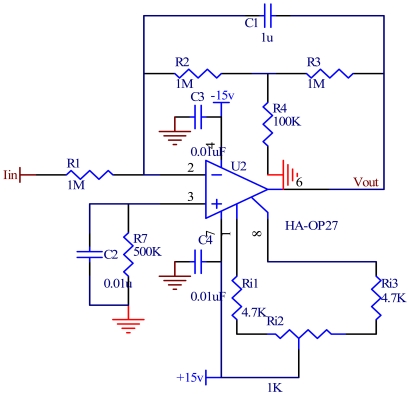
The integral T-style feedback I/V conversion circuit.

A vacuum microelectronic accelerometer emits electrons by cathode tip array, and then the electrons form a weak emission current. For weak acceleration, the current change value is only nA level, and it is sensitive to the emission voltage (an exponential relationship). The main problem of weak current detection is the noise and drift, so this paper focuses on a high-precision, low-noise I/V conversion. A low noise prepositive amplifier OP27 is chosen, and the maximum equivalent input noise voltage is 3.8 nV/rtHz at 1 kHz. But the amplifier will bring additional resistance thermal noise and active noise which are both random smooth noise, and in accord with Gauss' rule. An integral I/V conversion circuit will eliminate high frequency noises by integral effect. Otherwise, the large gain and high precision are needed in I/V conversion circuit, and the integral T-style feedback network is chosen, as shown in Figure 8. This I/V conversion circuit can use a smaller resistance to attain a higher input resistance and larger gain. It can achieve high-precision I/V conversion, as well as increase load capacity. Furthermore, it can depress high frequency noise, and reduce the influence on the change of bias voltage. Low frequency and DC current signal can directly go through the T-style network and be converted into voltage. While high frequency noise can be eliminated by the integral action of capacitance C_1_, and the noise infection will be controlled by the integration time [14]. R_7_ is a compensating resistance, which can eliminate temperature drift. C_3_ and C_4_ are decoupling capacitor used to reduce the interference of power lines, as well as prevent the amplifier from producing self-oscillation. R_i1_, R_i2_, R_i3_ are used to adjust the offset voltage of OP27. The I/V conversion voltage is given by:

(11) $$V_{\text{out}}=-R_{2}(\frac{R_{3}}{R_{2}}+\frac{R_{2}}{R_{4}}+1)I_{in}=-RI_{in}$$

As analysed in [15], the input noise of T-style feedback circuit is mainly determined by the resistor R3, and it is far smaller than the equivalent resistance R. So the noise voltage of T-style feedback circuit will be well controlled. The noise can be eliminated at the front of the circuit.

### 4.2. Differential Amplification

**Figure 9.**
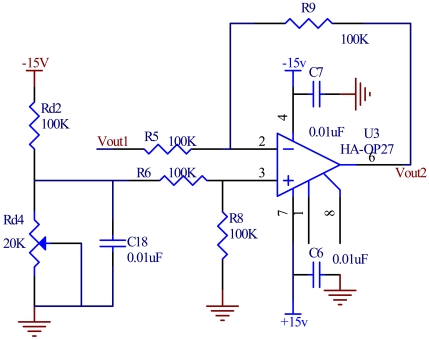
The circuit of differential amplification.

When a vacuum microelectronic accelerometer meets emission conditions, the tip array begins to emit electrons and then the electrons form an emission current, even if there is no acceleration. After I/V conversion, the deflection voltage appears. For precision control, a differential amplification is used to eliminate this deflection voltage. The differential amplification circuit is shown in Figure 9. The reference voltage V_ref_ is obtained by the distribution of high precision resistors.

### 4.3. Low-Pass Filter

**Figure 10.**
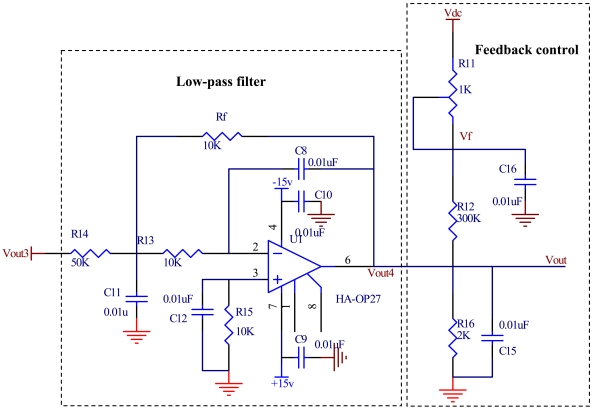
The circuit of low-pass filter and feedback control loop.

In the light of the modeling parameters analyzed by Matlab, the infinite gain multi-channel feedback 2-order low-pass filter is chosen, as illustrated in Figure 10. The transfer function is given by:

(12) $$\frac{-\frac{R_{f}}{R_{1}}(\frac{1}{R_{1}}+\frac{1}{R_{2}}+\frac{1}{R_{f}})}{s^{2}+\frac{1}{C_{1}}(\frac{1}{R_{1}}+\frac{1}{R_{2}}+\frac{1}{R_{f}})s+\frac{1}{C_{1}C_{2}R_{2}R_{f}}}$$

The denominator shows that, the filter can't generate self-oscillation, even if the pass band amplification is too large; the filter has good stability. Otherwise, the transfer function will correlate with Equation (10) by choosing appropriate resistors and capacitances. The low-pass filter not only effectively suppresses high-frequency noise, but also improves the dynamic performance of the accelerometer system.

### 4.4. Feedback Control Loop

For the sake of enhancing output linearity and dynamic response range of vacuum microelectronic accelerometer, the electrostatic force balance technology is adopted, and it forms a servo accelerometer. The feedback network uses linear technology which is constituted of resistors and capacitances, as shown in Figure 10. The output voltage of feedback control loop is given by Equation (13):

(13) $$V_{f}=\frac{R_{12}+R_{16}}{R_{11}+R_{12}+R_{16}}\times V_{dc}+\frac{R_{11}}{R_{11}+R_{12}}\times V_{\text{out}4}$$

where V_dc_ is the DC deflection voltage and V_out4_ is a negative output voltage of forward circuit. Thus a negative feedback control loop is obtained. It has two effects: first, it adjusts the distance between proof mass and anode plate, and makes the cathode tip array emit electrons; second, when there is acceleration acting on the proof mass, the feedback voltage *V_f_* changes, and results in an opposite electrostatic force. The proof mass will return to the balance position. Using linear technology makes the feedback system of vacuum microelectronic accelerometer simple.

In addition, a series of measures are adopted to reduce the interferences, such as accessing decoupling capacitor between power and ground, laying out ground wire reasonably, adding shielding enclosures while testing, and so on. We also utilize zero adjusting resistors to decrease zero drift. This circuit is consistent with the analysis results in Part 3.

## 5. Experiments

**Figure 11.**
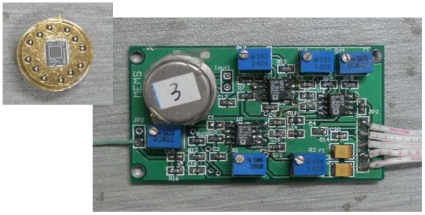
Photograph of accelerometer with signal processing circuit.

The simulation and debugging of signal detecting circuit are done. Figure 11 shows the photograph of vacuum microelectronic accelerometer with signal processing circuit.

**Figure 12.**
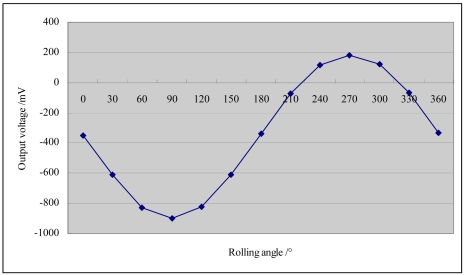
Output curve of static rolling experiment in ±1g gravitational field.

The static rolling experiment in ±1 g gravitational field is done. Figure 12 shows the output curve of the acceleration system. Where x-axis is the rolling angle and y-axis is the corresponding output voltage (mV). It illustrates that they satisfy a good sine relationship. The emission voltage is 2.478 V, and the feedback voltage is 5.112 V at 0 g input. A 12 points method is used during the testing, and the interval is 30 so that the accelerometer can be sensitive to different acceleration components [16].

**Figure 13.**
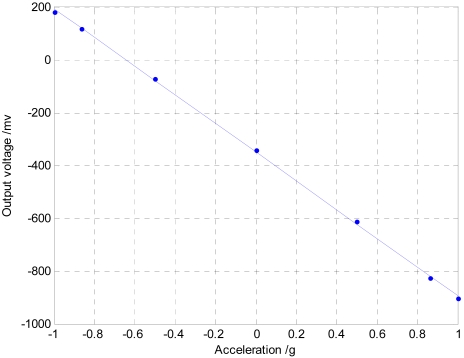
Output fitting curve of vacuum microelectronic accelerometer.

Figure 13 shows the least-square linear fitting curve of the output voltage and corresponding acceleration, and the linear equation is as follows:

(14) V=−349.955−0.543a

It shows that the output sensitivity is 543 mV/g, and the nonlinearity is 0.94 % in ±1 g measurement range.

**Figure 14.**
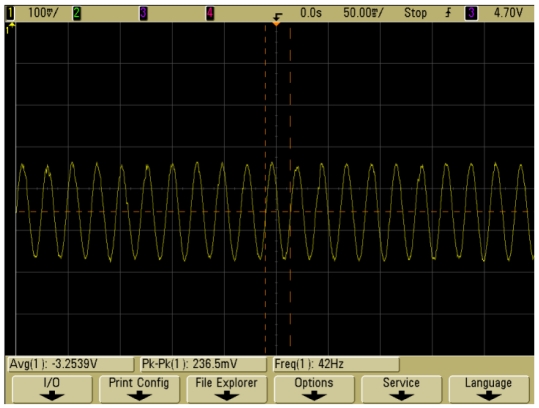
Output voltage of the accelerometer at 42 Hz, 0.5 g input sine acceleration.

Figure 14 illustrates the time history response of the output of the vacuum microelectronic accelerometer at 42 Hz when the input acceleration is 0.5 g. It shows that this accelerometer system has stable output and good performance.

**Figure 15.**
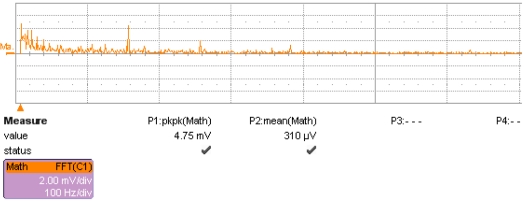
Noise spectrum density of vacuum microelectronic accelerometer.

The noise of the accelerometer was measured. Figure 15 shows the noise spectrum density with the signal detecting circuit showed together at 0 g position, where the x-axis is the frequency and the y-axis is the peak-to-peak noise spectrum density (mV/rtHz), and the maximal value is 4.75 mV/rtHz. After transfer to the input end, the noise level is 3 mg/rtHz. The low frequency noise plays a major role, showing a 1/f behavior. Other peak values appear at the integral multiple of industrial frequency noise, mainly brought by DC voltage. These noises will influence the resolution to a great extent, and must be decreased as much as possible. Further work must be done to realize higher resolution.

After verifying the fundamental performance of the vacuum microelectronic accelerometer, a rough comparison with a tunneling accelerometer is done. The deflection voltage of a tunneling accelerometer is a high DC voltage of 100 V, but in our design it's only several volts. Therefore the energy consumption will be greatly reduced. In general, the emission distance between the anode and cathode of tunneling accelerometer is only several angstroms. For a vacuum microelectronic accelerometer this distance can reach 0.1 μm, and the motion range of proof mass is broadened. Besides, a vacuum microelectronic accelerometer emits electrons by cathode tip array which contains thousands of single tips, so the output current will be much larger than tunneling accelerometer, and the signal detecting circuit is easier to be achieved.

## 6. Conclusions

For high sensitivity and good performance, the system analysis and a high precision signal detecting circuit of a vacuum microelectronic accelerometer were studied in this paper. Based on the structure and working principles, a simple but effective mathematical model is established. Matlab is used for system-level analysis. The step response and root locus of the system are also studied and discussed. A 2-order low-pass filter is needed in the circuit so that the accelerometer system has good performance. Otherwise, in order to attain high precision and good linearity, weak signal detecting theory and closed-loop control mode are adopted. Compared to previous examples, this circuit is simply and reliable. Experiments are carried out to verify the characteristics of our vacuum microelectronic accelerometer system. The results show that the output sensitivity of vacuum microelectronic accelerometer is 543 mV/g, and the nonlinearity is 0.94 % in ±1 g measurement range. Work on an application specific integrated vacuum microelectronic accelerometer circuit is underway. The system noise will be evidently reduced and the performance of accelerometer will be improved. Other experimental results will be reported when they become available.

## Figures and Tables

**Table 1.** Design parameters of vacuum microelectronic accelerometer.

Design parameters	Values
Mass, *m*	904 μg
Spring constant, *k*	88 N/m
Damping coefficient, *b*	6.58×10^-3^ N·S/m
Feedback coefficient, *K_f_*	3.39×10^-5^ N/V
Static emission current, *I_0_*	0.1 μA
